# Electromagnetic Torso Scanning: A Review of Devices, Algorithms, and Systems

**DOI:** 10.3390/bios11050135

**Published:** 2021-04-27

**Authors:** Sasan Ahdi Rezaeieh, Amin Darvazehban, Azin S. Janani, Amin M. Abbosh

**Affiliations:** School of ITEE, The University of Queensland, Brisbane 4072, Australia; a.darvazehban@uq.edu.au (A.D.); azin.janani@uq.edu.au (A.S.J.); a.abbosh@uq.edu.au (A.M.A.)

**Keywords:** torso scanning, antennas, processing algorithms, electromagnetic imaging

## Abstract

The past decade has witnessed a surge into research on disruptive technologies that either challenge or complement conventional thoracic diagnostic modalities. The non-ionizing, non-invasive, compact, and low power requirements of electromagnetic (EM) techniques make them among the top contenders with varieties of proposed scanning systems, which can be used to detect wide range of thoracic illnesses. Different configurations, antenna topologies and detection or imaging algorithms are utilized in these systems. Hence, to appreciate their progress and assess their potential, a critical review of EM thoracic scanning systems is presented. Considering the numerous thoracic diseases, such as fatty liver disease, lung cancer, respiratory and heart related complications, this paper will exclusively focus on torso scanning systems, tracing the early foundation of research that studied the possibility of using EM waves to detect thoracic diseases besides exploring recent progresses. The advantages and disadvantages of proposed systems and future possibilities are thoroughly discussed.

## 1. Introduction

The urge and curiosity of human beings toward understanding diseases and developing tools to diagnose them can be backdated to ancient times and has been an ever-developing part of science throughout past centuries. However, it was the invention of the X-ray by Wilhelm Rontgen in the 19th century that created a new direction in medical science. It provided a third eye to the medical staff that could confirm or reject their hypothetical diagnosis. This advancement has changed the course of treatments and raised medical standards significantly. Hence, huge investments were made to enhance this technology and improve the quality of the obtained images, leading to the invention of Computed Tomography (CT scan) in the second half of 20th century. However, despite all these innovations, both systems came with an undesirable caveat: they use ionizing radiation. Efforts by numerous researchers resulted in the invention of magnetic resonance imaging (MRI) that utilizes a combination of a strong magnetic field and electromagnetic (EM) waves to map the changes inside human body. MRI is accepted as the gold standard among medical imaging devices and its image quality has significantly improved, thanks to advancements in imaging algorithms and coil fabrication technology. Consequently, MRI technology comes with a huge price tag and highly shielding requirements to contain the strong magnetic fields. Hence, it is not suitable for rapid onsite diagnosis and frequent monitoring, besides limiting its accessibility to large medical centers.

Motivated by the aforementioned limitations, researchers have been investigating alternative or complementary techniques that are non-invasive, safe, low cost and portable. EM techniques are among the top candidates that have been widely studied. The basis of using those techniques is the fact that characteristics of EM waves, such as phase and magnitude, are altered by the dielectric properties of biological tissues [1]. For instance, a cancerous cell has higher fluid content compared to a healthy one [2]. This results in a change in the response of EM signals, and this change can be potentially exploited for detection purposes. This review focusses on application of EM scanning systems for thoracic diseases, as one of the main contributors to mortality rates in the world [3]. Fluid accumulation inside (pulmonary edema) and around (pleural effusion) the lungs is the common symptom for various diseases such as heart failure, lung cancer, breast cancer and more recently COVID-19 [4,5,6]. Hence, the ability to detect accumulated fluid at early stages can potentially lead to early diagnosis of the underlying diseases. The possibility of detecting lung fluid was first proposed by Susskind in 1973 [7], where the use of a combination of a cathode ray tube and an antenna was proposed as a scanning platform. The first studies on the application of microwaves to detect pulmonary edema were performed by Pedersen [8,9]. These studies modeled human body as a load and calculated reflection coefficient based on the changes inside the torso. Hence, those studies concluded that any changes inside the torso affect the magnitude and phase of EM reflection and transmission coefficients, although only limited tests were performed on the magnitude of those signals. To further improve these outcomes, two methods were later introduced in 1983 by Iskandar et al. [10] to measure the variations in lung water. The first method analyzed the phase of transmission coefficient of a microwave applicator [11], whereas the second method employed the radiometry concept that monitors changes in microwave emission levels [12]. To provide a more robust detection process, dielectric properties estimation methods were used to detect pathological changes in tissues inside lungs [13]. It follows the same logic that accumulated fluid inside or around lungs alters the average permittivity as experienced by the sensing antennas.

While all these methods have advanced the field, they all have limitation in that detection is performed based on the assumption of known dielectric properties of the healthy status of the scanned subject [8,9,10,11,13], which is difficult to achieve in practice. Accordingly, different approaches such as the combination of microwave transmission with X-ray scanning were investigated. The method in [14] estimated the amount of water content inside lungs using a method of moments. This study was an important step in moving towards a more sophisticated analysis of the torso’s internal tissues. This view was further advanced by the introduction of radar-based microwave imaging [15,16,17,18,19,20,21] and tomography methods [22,23], where the scattered fields from the abnormal tissue inside the torso is calculated using forward and backward processing methods. Considering the higher water content, in the case of edemas and cancerous tissues, the reflected/transmitted signals from those pathologies create a strong scattered field or high contrast permittivity map that can be shown as two- or three-dimensional images. The advances in computational methods and artificial intelligence open new horizons in research that were not feasible before. For example, a statistical analysis was adopted to predict changes in fat levels inside the liver [24]. This method is based on the high symmetry between the right and left sides of the body and analysis of the correlation between signals from these areas at several frequency points. In another method, the feasibility of a supervised decent method [25] for torso imaging was adopted to estimate the torso’s structural information, paving the way for real time imaging.

To ascertain the importance of the abovementioned advances, the present review focuses on torso scanning systems aimed at detecting thoracic diseases and thus does not include vital sign monitoring applications. Section 2 of the paper reviews electromagnetic scanning systems and discusses their operation principles. It then investigates different data acquisition methods and their role in complexity and accuracy of the system. Moreover, it reviews the most utilized scanning platforms for torso scanning systems, their capabilities and limitations. The safety considerations for torso scanning systems are thoroughly investigated in Section 3. The safely limits are investigated using specific absorption rate (SAR), followed by a discussion on range of SAR values for current EM torso scanners. Section 4 presents an overview of design criteria for torso scanning systems and provides a detailed review of two different categories of on-body matched and free space antennas. The advantages and disadvantages of each subcategory in terms of penetration, impedance matching, size and fabrication complexity are investigated. Section 5 classifies the utilized algorithms in these systems into three subcategories of detection only, classification and detection and localization (imaging). This section concludes that there is a compromise between accuracy of the scanning system, its complexity, and practicality. Each section is accompanied by a comparison table that highlights the pros and cons of each subcategory. Section 6 offers conclusions based on the discussions provided in previous sections and provides thoughts for future development of these systems.

## 2. Electromagnetic Scanning Systems

An electromagnetic scanning platform includes two main elements: (1) hardware and (2) software. In the hardware unit, antennas are used to transmit signals towards torso (imaging domain) and receive the reflected or transmitted signals that are then recorded using a vector network analyzer (VNA), or any proper EM transceiver, and stored in a computational tool that contains the detection algorithm (software). The detection algorithm analyzes different aspects of the signal and either form an image or give estimation of the pathology. While the next sections of the paper will go through details of utilized elements and methods, this section focuses on the overall operating mechanism and formation of EM scanning systems.

### 2.1. Data Acquisition Methods

Data acquisition methods can be broadly categorized as mono-static and multi-static ones. In a mono-static system the same antenna is utilized as the transmitter and receiver. Therefore, these systems can only analyze the reflection coefficient signals [26,27,28]. Since the system has no switching requirement, its implementation is simple. However, due to the lack of transmission coefficient data, the system has less degree of freedom to compensate for the effect of noise on detection decision. Additionally, the system is less sensitive to changes in the dielectric properties of deeper targets. Hence, this method is more suitable for surface or subsurface abnormalities [29]. In the multi-static system, an array of antennas, at least two, are used to transmit and receive signals [24,30,31,32]. This process continues sequentially until all the antennas in the array have received signals from each other. Hence, more data in the form of transmission coefficient signals between different channels is obtained compared to a mono-static method. This enables higher detection accuracy and enhances deep target detection. However, the system requires a switching network, which introduces additional insertion loss to signals plus increasing the scanning time [33,34].

### 2.2. Scanning Platform

**Figure 1:**
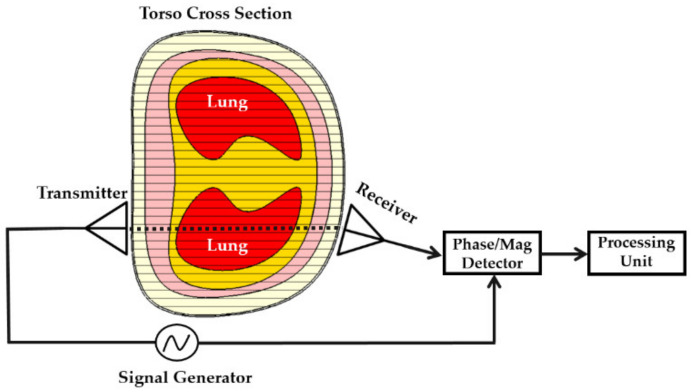
Configuration of an early torso scanner setup (concept taken from [31]).

Two main factors define the configuration of the scanning platform: (1) requirements of the detection algorithm and (2) size of the antennas. Detection algorithms are divided between the ones that focus on detection only and the ones that aim at detection and localization of the malignancy. The size of the antenna mainly depends on the operating frequency band and this could be a limiting factor when operating at low microwave frequencies. Generally, algorithms that only aim at making a binary decision of presence or absence of the malignancy, e.g., water accumulation, require simpler setups. As seen from Figure 1, the system that is used in early studies [31] follow this approach and use two applicators on either side of the torso to capture and utilize transmitted signal. A proper detector is used to measure the phase of the transmitted signal, which is then recorded using a VNA.

The second category of algorithms requires more sophisticated scanning platforms with higher number of antennas. To provide a statically quantifiable data or map the location of the malignancy, several scans from different angles and positions around the torso are required. To fulfill the requirements of these algorithms, three main categories of scanning platforms are designed: (1) linear platforms, (2) circular platforms and (3) quasi-circular platforms.

#### 2.2.1. Linear Platforms

**Figure 2:**
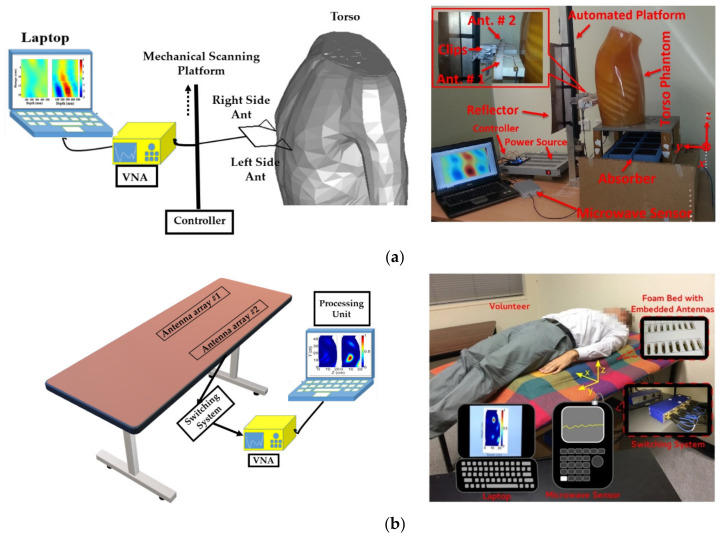
Linear scanning platform. (**a**) Dynamic system, schematic view (left) and system view (right). Reproduced with permission from [35]. Copyright 2014 IEEE. (**b**) Static system, schematic view (left) and system view (right). Reproduced with permission from [18] (open access).

Linear platforms are designed to scan the rear side of the torso and are generally used with algorithms that utilize differential detection approach [18,26]. In these systems an antenna or an array of antennas scan the right and left sides of the torso and their location is mechanically displaced along the torso [18,35]. Stepper motors are used to perform the movement with small, around 1 mm, steps at each scan. To simplify the scanning setup, a mono-static data acquisition technique can also be utilized in these systems. An example of these systems is depicted in Figure 2, where two antennas are located side by side on a lever to eliminate positioning errors while being displaced up and down. The main advantage of this scanning system is that high number of scans can be performed, and hence the obtained image could provide an accurate estimation of the location of the malignancy. However, these systems come at the cost of more complicated setup and slower scanning process.

The second types of linear systems are the static ones, where antenna elements form an array and are fixed in a position along the torso. Examples of the systems are presented in [18,34], where two arrays of antennas are embedded in a foam or a bed for a patient to lay on when the scan is performed. These platforms remove the complications with the movable systems at the cost of using lower number of antennas due to mutual coupling considerations. To compensate for reduced antenna numbers and to maintain the accuracy of the system, a multi-static data acquisition method is utilized. Hence, a switching network is added as part of the system, and this in turn increases the overall cost of the scanning system (See Figure 2b)

The linear scanning systems are most suitable for applications where scans of the upper sides of the torso are required. This facilitates a uniform scan for all male and female subjects and the obtained signals are not convoluted by the presence of breast. However, due to limited power allowed in EM scans, these systems are less sensitive to deep malignancies or the ones that are far away from the antennas.

#### 2.2.2. Circular Platforms

**Figure 3:**
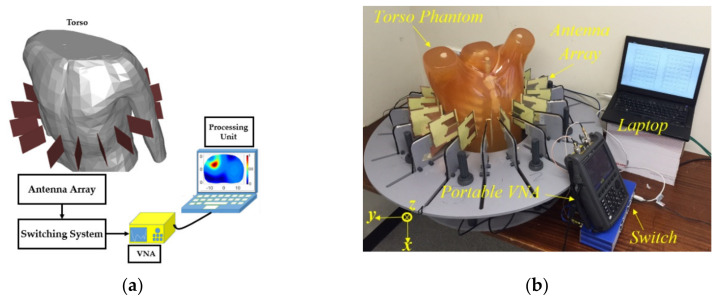
Antenna array platform for scanning the torso area: (**a**) schematic view; (**b**) system view. Reproduced with permission from [36]. Copyright 2016 IEEE.

To alleviate the problem of low accuracy for deep targets, circular platforms are proposed in which the antennas are located around the torso [36,37]. An example of a circular platform configuration is depicted in Figure 3. The system consists of an array of 16-element antennas, a VNA and a laptop as the processing unit. A monostatic data acquisition method is performed where each of the antennas are used to both transmit and receive backscattered signals. These setups are used for imaging algorithms that require obtaining information from all angles around the object. This is necessary to produce a scattering profile or map dielectric properties across the imaging domain, e.g., torso. Three dimensional images can also be obtained by locating the circular array on a moving flange [29] and scanning different heights across the torso. Both mono-static and multi-static data acquisition techniques can be used to obtain the scattered signals. The main consideration in these systems is the distance of the antenna from the torso, due to semi-elliptical shape of the body. Considering the significant variations between torso sizes in different individuals, the designed systems are generally not fit for every single case. While a certain degree of movement for antenna locations is allowed within the system, a fixed distance between the antenna and body cannot be guaranteed/achieved for all scanning cases, hence, creating difficulties for the imaging algorithm in the form of ghost targets due to stronger reflections at the skin/air boundary. This is addressed by using strict calibration measures before each test, besides filtering techniques that add to the complexity of the system. The circular system setup is best suited for applications where information regarding the location, size and severity of the malignancy is required.

#### 2.2.3. Quasi-Circular Platforms

**Figure 4:**
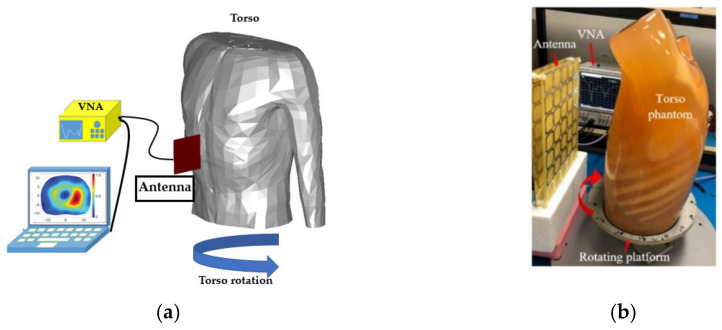
Quasi circular platform: (**a**) fixed beam antenna (concept taken from [19]). (**b**) Pattern reconfigurable antenna. Reproduced with permission from [38] (Copyright 2019 IEEE).

While circular systems provide a convenient setup for scanning, their design can be complex due to high number of antennas and switching network that ensures timely scanning of the patient. Therefore, a simpler version of these systems has also been utilized, in which the system is comprised of a single antenna and the subject is located on a turning platform [19]. In this system the subject is rotated at desired intervals, e.g., every five degrees, and the antenna constantly scans the patient until a full scan is performed. This system can only operate in mono-static data acquisition mode and the imaging algorithm is limited to the use of reflected signals only. However, a higher number of scans/data points are obtained that can potentially compensate for the lack of transmission coefficient data. The configuration of the proposed system is depicted in Figure 4.

**Figure 5:**
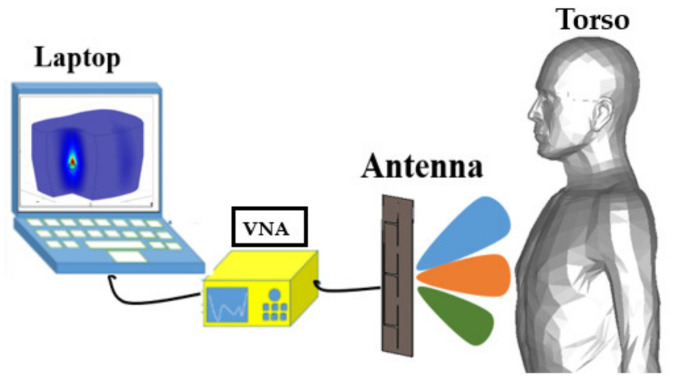
Schematic of pattern reconfigurable antenna for torso scanners.

Additionally, three dimensional scans can also be obtained using pattern reconfigurable antennas [38]. An example of a quasi-circular platform using pattern reconfigurable antenna is shown in Figure 4. The schematic of pattern reconfigurable antenna for torso scanning is depicted in Figure 5.

#### 2.2.4. Wearable Platforms

**Figure 6:**
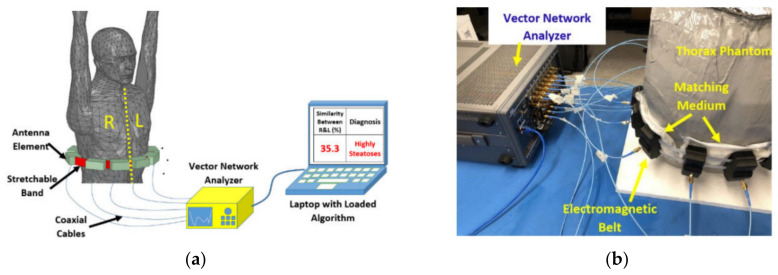
Wearable platform: (**a**) Schematic view; (**b**) system view. Reproduced with permission from [24] (open access).

To overcome the complications of finding the exact location of the antenna with respect to the body, wearable systems can be used [13,24,39,40]. As shown in Figure 6, in these systems the antenna array is located on the body, and depending on the utilized detection algorithm, the antennas can be located locally adjacent to the subject [40] or surround the circumference of the imaged body [24]. These systems can utilize multi or mono static data acquisition methods and can accommodate larger number of antennas as antenna size can be significantly reduced due to the body loading on the antenna. This arrangement provides the scanning system with larger number of data points that can be collected during the scan.

## 3. Safety Considerations

Electromagnetic medical scanning systems should follow the safety regulations that are defined by different government bodies such as Federal Communications Commission (FCC) [41] in United States and European Council (EC) [42] in Europe. The safety limit is specified using specific absorption rate (SAR), which is the amount of energy stored in human body during each exposure. These limits vary depending on different jurisdictions and are generally defined as Watts per kilogram. For instance, FCC limits maximum exposure at 1.6 W/kg, whereas EC allows exposures up-to 2 W/kg.

Two main factors affect the obtained SAR value in an EM scanning system; (1) the operating frequency band and (2) the distance of the antenna from body [43]. The operating frequency of the antenna can significantly affect the obtained SAR value as it has an inverse relation with the operating wavelength. Consequently, lower microwave frequencies have deeper penetration that result in higher SAR values [44]. Similarly, SAR value has an inverse relation with the distance of the antenna from human body. This is attributed to a wider distribution of EM field at longer distances compared to the focused distribution at closer distances [43]. Studying torso scanning systems reveals that using a 1 mW (0 dBm) transmitted power results in SAR values between 0.004–0.04 W/kg at 0.6–0.9 GHz [18,24,29,33,34]. Lower values are obtained when antennas are located farther from body [29,33] and higher values are attributed to antennas closer to body [18,24,34].

## 4. Antenna Designs

The effectiveness of EM scanning system for the detection of different types of diseases highly depends on performance of the utilized antennas. A review of some important antennas that are utilized for medical EM scanning systems, their operation principles and performance requirements are presented in this section.

### 4.1. Antenna Design Criteria

The following main requirements should be considered for designing antennas for EM scanning systems: operating frequency, bandwidth, and radiation characteristics, such as directivity, penetration level and efficiency.

**Figure 7:**
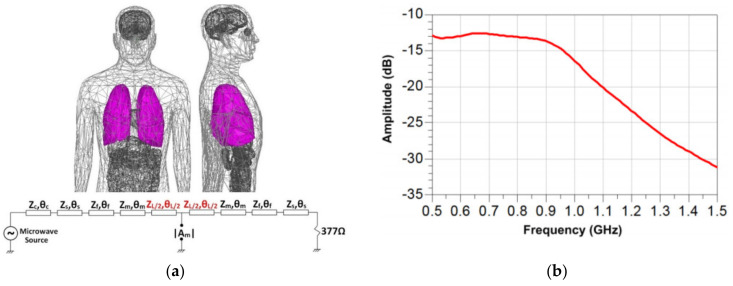
Signal penetration into human torso: (**a**) lumped element model of human torso; (**b**) variation of signal magnitude at the center of human lungs with frequency. Reproduced with permission from [46]. Copyright 2013 IEEE.

The first step in designing antennas for EM scanning systems is to define the optimum frequency bandwidth for the scanning system. Human tissues have frequency-dependent dielectric constant (relative permittivity) that generally decreases with increasing the operating frequency. Moreover, human tissue is a lossy medium with losses that increase with frequency [45]. Hence, increasing the operating frequency in general decreases signal penetration into human tissues. Studying the literature reveals that historically low microwave frequencies at 0.9 GHz have been utilized for torso scanning applications [8,9,10,11,30,31,32] that is consistent with signal loss considerations. However, to define a range for wideband torso imaging/detection applications, a study is conducted in [46], where the equivalent circuit model of an average human torso is utilized. The variations of signal magnitude at the center of the lungs for different frequencies are calculated (Figure 7). As seen in Figure 7b the attenuation of the penetrated signal increases significantly at frequencies above 1.5 GHz. Moreover, there is a direct relation between antenna’s physical size and operating wavelength. Microwave frequencies below 0.5 GHz lead to physically large antennas, which makes the EM scanning system bulky and hinders its portability. Additionally, decreasing the operating frequency adversely affects resolution of the obtained images [47]. Considering all these factors, recent studies indicate that using the operation bandwidth of 0.5–1.5 GHz provides the best compromise between the signal penetration into human torso, image resolution, and antenna size [15,30,48].

Different studies reveal that the accuracy of radar-based imaging algorithm is directly related to the utilized bandwidth of the antenna. The study in [49] reveals that in radar-based EMI systems, the lack of discrete observation points can be compensated using different scattered profiles from frequency samples across a wideband signal. Hence, wideband signals are preferred in EM scanning systems [18,19,20,50].

**Figure 8:**
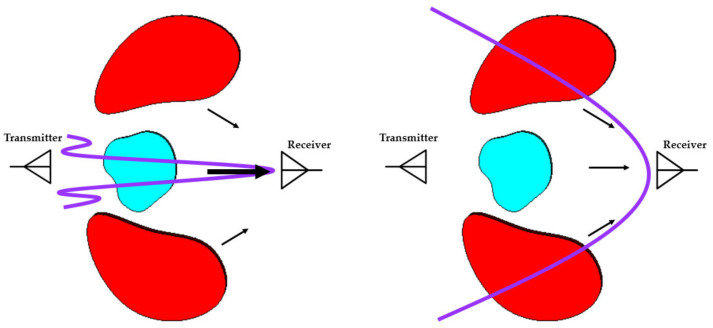
EM scanning system using focused beam antennas (left) and non-focused beam antennas (concept taken from [51]).

The other important requirement of antenna design for EM scanning systems is focused beam radiation in near field and/or far-field. In EM imaging systems, unidirectional radiation is preferred to reduce the adverse effects of environmental noise and scattered fields on signal-to-noise ratio, hence providing better detection results [51,52]. The effects of the focused beam antennas on the reconstructed images in EMI systems are thoroughly investigated in [51]. As seen from Figure 8, unidirectional focused beam antennas can reduce the adverse effects of undesired scatterers, such as surrounding organs in the imaging domain. A performance comparison of the unidirectional and omnidirectional antennas for the EMI system is presented in [52]. The results indicate that the unidirectional antennas have significantly better performance in reducing the artifacts that result in higher resolution/accuracy images.

### 4.2. Antenna Categories

Investigating EM scanning systems reveals that two broad categories of radiators are used to scan the human torso; (1) on-body matched antennas [24,30,31,53,54], and (2) free-space antennas [55,56,57,58,59,60]. Different methods were proposed to design these antennas that are detailed in this section.

#### 4.2.1. On-Body Matched Antennas

An on-body matched antenna is designed in the presence of human body model and its performance is optimized considering the frequency-dependent characteristic of investigated body area, e.g., torso. This ensures that the radiated power by the antenna is directly penetrated inside the body and no reflection occurs in the air-body boundary. Because these antennas are located on-body, their performance is not measured using conventional measurement concepts such as directivity or far-field radiation patterns. Instead, their success is measured by the intensity of electromagnetic field that is induced/directed inside investigated area. The EM field intensity can be increased by optimum impedance matching and directing the radiated EM signal towards the body. Considering the frequency dispersive properties of human tissues, wideband impedance matching is a challenging issue.

**Figure 9:**
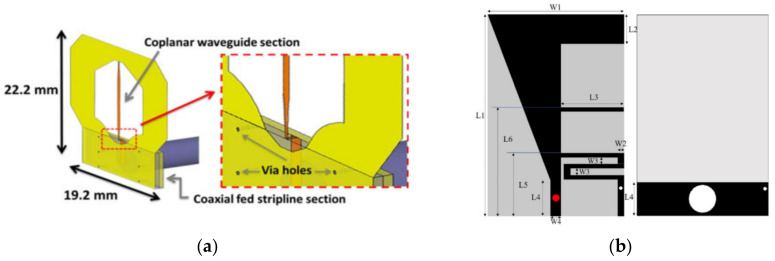
On-body matched antenna: (**a**) coplanar waveguide-type antenna (reproduced with permission from [30], Copyright 2014 IEEE); (**b**) L-shaped monopole antenna (reproduced with permission from [40], Copyright 2020 IEEE).

The most straight forward method to achieve impedance matching is to terminate the antenna with a resistor. The design of these antennas can be traced back to early studies where an electromagnetic coupler was used as microwave sensors to transmit electromagnetic signals into the torso and receive reflected/transmitted signals [30,31,32,55]. These couplers are designed based on co-planar waveguide structures. A strip-line impedance matching transition between coaxial cable and coplanar waveguide is implemented to increase their operating bandwidth as depicted in Figure 9a [30]. These structures are terminated by resistors to provide impedance matching to human body. However, the addition of the resistor decreases the radiation efficiency of this antenna/coupler significantly, hence, it reduces their practicality for deep target detection.

To overcome the limitations in obtaining matching with resistor termination, different methods have been developed. An L-shaped monopole antenna is proposed to reduce the size of the antenna by increasing its electrical length [40]. The configuration of the antenna is depicted in Figure 9b. As seen, a meandered shorted stub and open-ended stub are used to adjust the two main operating modes of the antenna. To enhance the bandwidth of the antenna, a triangular patch is added to the L-shaped monopole. A combination of a rectangular ring-shaped monopole and a meandered patch is proposed in

Two sets of L-shaped and I-shaped slots are etched to the ground plane to create new resonances at higher frequencies and improve the operating bandwidth [53].

**Figure 10:**
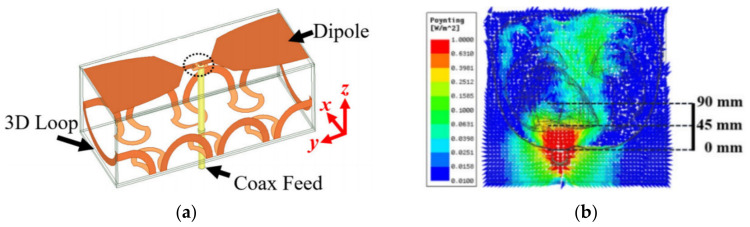
Hybrid loop-dipole antenna: (**a**) configuration of the antenna; (**b**) signal penetration. Reproduced with permission from [24] (open access).

Despite their success in achieving wide operating bandwidth, the utilized antennas possess monopole-type radiation characteristics. Consequently, while part of the accepted power is matched to the torso, the rest of the power is dissipated from the back of the antenna. To address this problem, hybrid loop-dipole structure is proposed in [24], where the theory of complimentary antennas is utilized (Figure 10a). This method utilizes the well-known fact [61,62,63,64] that if the loop and dipole antennas are excited simultaneously with equal phase and magnitude, the resulting radiation pattern would be unidirectional with suppressed back lobe radiation, therefore, enhancing signal penetration inside torso by almost two-fold compared to a monopole structure [24]. The electric field penetration of the proposed design is depicted in Figure 10b.

**Figure 11:**
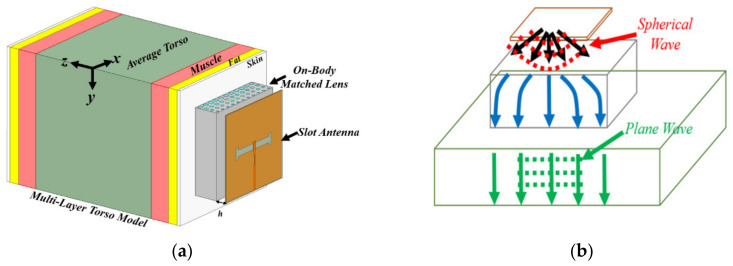
On-body matched GRIN lens: (**a**) configuration of the antenna; (**b**) spherical wave to plane wave transformer. Reproduced with permission from [54], Copyright 2021 IEEE.

**Figure 12:**
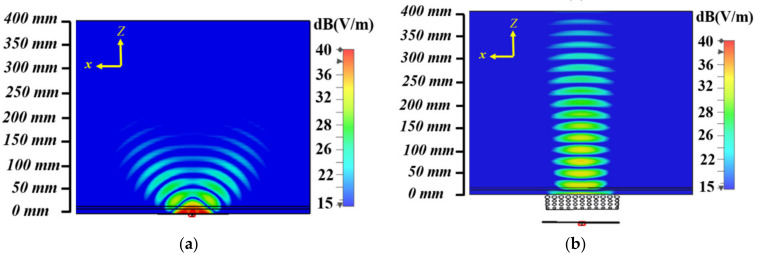
Electric field comparison: (**a**) on-body matched slot’; (**b**) on-body matched GRIN lens. Reproduced with permission from [54], Copyright 2021 IEEE.

Most radar-based imaging algorithms assume that the incident wave is a planar wave. This assumption is not fulfilled when using conventional body matched antennas that possess spherical wave-front for EM inside human torso. Consequently, the obtained images using these antennas are accompanied by errors regarding location of the malignancy, its size and formation of ghost targets due to the plane wave assumption of utilized algorithms. To address this problem, a body matched graded index (GRIN) lens antenna is proposed in [54] and depicted in Figure 11. This lens, which is based on the theory of multi-layer structures, transforms the spherical wave from its exciting source (slot antenna) to a planar wave inside the torso as shown in Figure 11b. This is achieved through transitioning wave from air that has a low permittivity of 1 to that of an average torso, 45, through gradual increase in permittivity in each lens layer. To fabricate different dielectric layers, a combination of low permittivity plastic and water is utilized. To obtain the desired dielectric values, a host structure that has different hole sizes is 3-D printed using the plastic material, and then filled with water. The obtained results reveal that the antenna achieves a strong penetration (more than 6 dB) compared to the body matched antenna (see Figure 12).

#### 4.2.2. Free-Space Antennas

To accommodate the requirements of different detection algorithms, free-space antennas were proposed and widely used to build alternative platforms. Free-space antennas are designed without the presence of body and their radiation properties are characterized using directivity, gain and radiation patterns. The detection is performed using the differences in the reflection and transmission coefficients in free space and in front of human body. These antennas strive to have compact sizes, wide operating bandwidth, and high gain/directivity. Considering that torso scanning systems operate at low microwave frequencies, obtaining all these merits is a challenging task. Hence, there will always be a compromise that is defined by the scanning system. This section summarizes the proposed designs.

**Figure 13:**
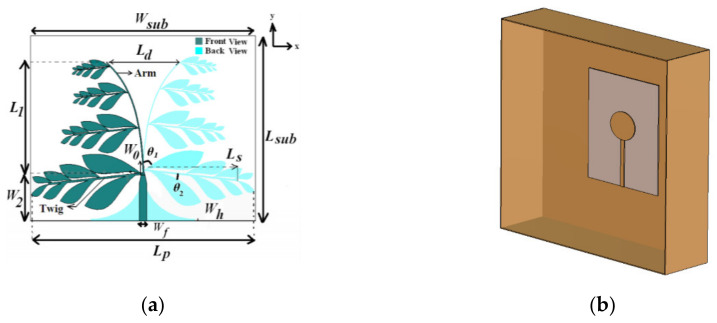
Free-space antenna: (**a**) fractal leaf Vivaldi antenna (reproduced with permission from [66], Copyright 2017 IEEE); (**b**) cavity-backed elliptical monopole antenna (concept taken from [71]).

Vivaldi antennas have been widely used in EM scanning systems due to their wideband operation and high radiating gain [28,55,56,65,66,67]. These antennas are generally comprised of a strip feed that is magnetically coupled to a flared ground plane. Corrugation methods [67,68] and fractal leaf arm techniques [56,66] are applied on the flared ground structure to increase the electrical length of the antenna for a broad bandwidth and high gain. An example of a Vivaldi antenna with fractal leaf arm is depicted in Figure 13a. Vivaldi antennas have been investigated for pulmonary edema detection [55] as well as lung tumor detection [56].

Owing to their unidirectional radiation pattern, patch antennas are widely used in some EM scanning systems [42,43]. In [69,70], dual patch antennas are used as electromagnetic sensors for pneumothorax diagnosis applications. Monopole antennas are used for lung tumor detection applications [44,45]. To improve signal penetration, the monopole antenna structure is loaded with a cavity back to achieve unidirectional radiation and hence increase the signal penetration into the human chest [71]. An example of cavity-backed elliptical monopole is illustrated in Figure 13b. Similar to on-body matched antennas, a combination of a loop and dipole can be used to increase directivity of the antenna and reduce its back radiation [24,36,57].

To overcome the size constraints that are imposed by the limited torso area, folding techniques are used in EM scanning antennas to reduce the physical size of antennas and increase the operating bandwidth and antenna’s directivity [26,58,72,73]. The process involves folding the edges of a planar antenna to form three dimensional structures. This method reduces the back radiation of antenna by changing the current flow alongside the antenna’s edge which eventually reduces its size. A three-dimensional folded loop-monopole structure for EM scanning systems is presented in [57]. The loop-dipole composite is first designed based on a planar structure and then folded over an optimal folding line to increase the antenna’s directivity and reduce its size. Another wideband folded antenna operating in the frequency bandwidth of 0.77–1 GHz for the early-stage detection of congestive heart failure (CHF) is presented in [26].

**Figure 14:**
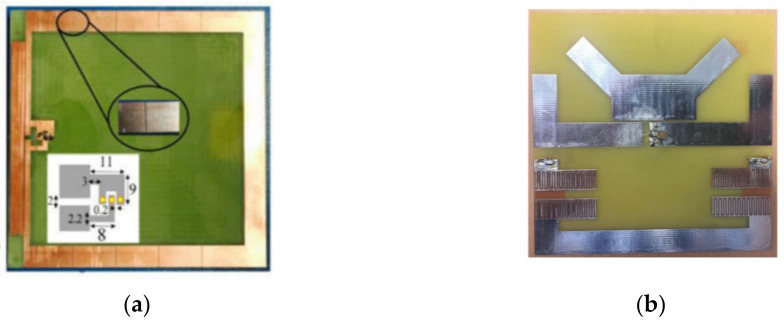
MNG loaded antennas: (**a**) MNG loaded loop antenna (reproduced with permission from [60], Copyright 2016 IEEE); (**b**) MNG loaded reflector Yagi-antenna. Reproduced with permission from [75]. Copyright 2017 IEEE.

Metamaterial techniques are also applied to reduce the size of antennas and improve directivity at low microwave frequencies. The structures are formed by applying series capacitance and/or shunt inductance to the host antenna to tune the resonance frequency. For example, mu-negative (MNG) metamaterial unit-cells were applied to a conventional square loop antenna in [60,74] to lower the resonance frequency of the antenna (Figure 14a). Since this resonance is independent of the antenna size, the overall size of the antenna is miniaturized by more than 50%. Additionally, it is shown that by non-periodic distribution of unit-cells, a unidirectional radiation pattern can be obtained. MNG unit-cell loading is also used to enhance the operating bandwidth of directional Yagi-antennas trough excitation of mu zero resonance below existing resonance of the Yagi antenna [75,76]. An example of MNG loaded reflector Yagi-antenna is illustrated in Figure 14b.

As stated before, certain detection algorithms require scanning different locations along the torso to perform comparative detection decisions. While mechanical movement is used to scan high number of regions, certain comparative algorithms can provide detection decisions using low number of torso regions, such as three in [29]. To eliminate the complications of mechanical movements, these systems can be fabricated using pattern reconfigurable antennas to electronically scan the upper, middle and lower parts of the torso [38,54,59,77,78,79]. A wideband reconfigurable loop antenna operating at 0.8–1.15 GHz for torso scanners is presented in [77]. Capacitive gaps are created along the loop arms to form virtual dipole arrays, which leads to unidirectional radiation with a peak gain of 2.1 dBi. Changing the location of gaps changes the direction of the dipole array, which leads to changes in the direction of the beam. Another pattern reconfigurable loop-dipole antenna with the capability of scanning the upper, middle and lower parts of an average human torso for pleural effusion detection is presented in [78]. The antenna consists of a one-wavelength loop, a have-wavelength bow-tie dipole, and two parasitic directors. The combination of loop-dipole mode increases the antenna’s directivity. The antenna operates in a wide fractional bandwidth of 55% at 0.8–1.4 GHz with a peak gain of 5 dBi. The beam steering is achieved using parasitic directors to alter the current distribution on the loop and enable beam switching in different directions to scan the whole chest area.

To satisfy the plane wave radiation assumption inside the imaging domain for radar-based imaging applications [80], reconfigurable metasurface lenses are proposed to achieve near field beam focused plane wave radiation inside the imaging domain [38,54,59]. Metasurface structures are built using an array of unit-cells that are distributed periodically and illuminated using a source antenna, e.g., a slot antenna. These unit-cells collimate the incident field into a focused transmitted beam. A pattern of reconfigurable metasurface antennas based on the offsetting technique is presented in [38]. Three half-wavelength microstrip-fed slots operating in the frequency band of 0.9–1.2 GHz radiate a metasurface layer. Beam steering is performed based on the excitation of each metasurface unit cell with different phase delays by changing the activated slot.

Pattern reconfigurability in metasurfaces can also achieve using programable unit cells by changing the characteristics of unit cells. A programable pattern reconfigurable metasurface for pulmonary edema detection is proposed in [59], where a metasurface layer containing 5 × 5 programable square ring resonator as the superstrate layer on an H-shape slot radiator is designed. Four PIN diodes are embedded in each cell to alter the electric field intensity within the metasurface layer and consequently switch the high intensify electric field in different directions inside the human torso.

**Figure 15:**
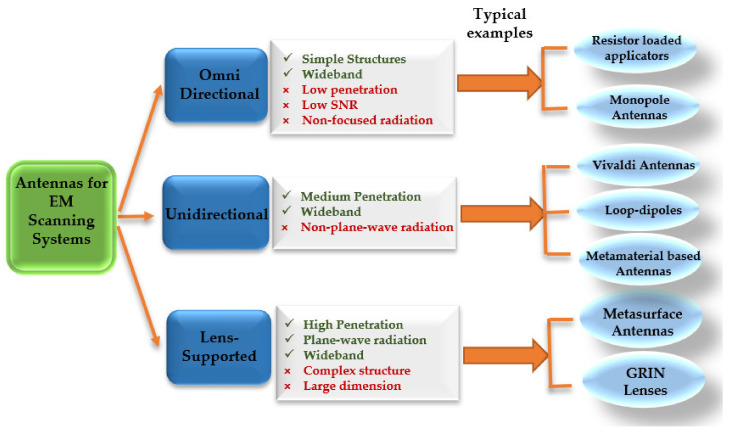
Characteristics of different types of antennas used in EM thoracic scanning systems.

Figure 15 summarizes and compares various types of antennas used in EM scanning systems to detect or localize any abnormality. Based on the requirements of different imaging/detecting algorithms and the required level of signal penetration into the human torso, a suitable antenna type is selected.

## 5. Microwave Detection Techniques

EM torso scanners utilize the high dielectric contrast between healthy and diseased tissues for detection purposes. Generally, the processing unit exploits any changes in the phase and/or magnitude of transmitted EM signals for abnormality detection or localization. The variation in dielectric properties of tissues along the wave’s propagation path alters the wave speed that results in the changes in phase and/or magnitude of the transmitted wave.

**Figure 16:**
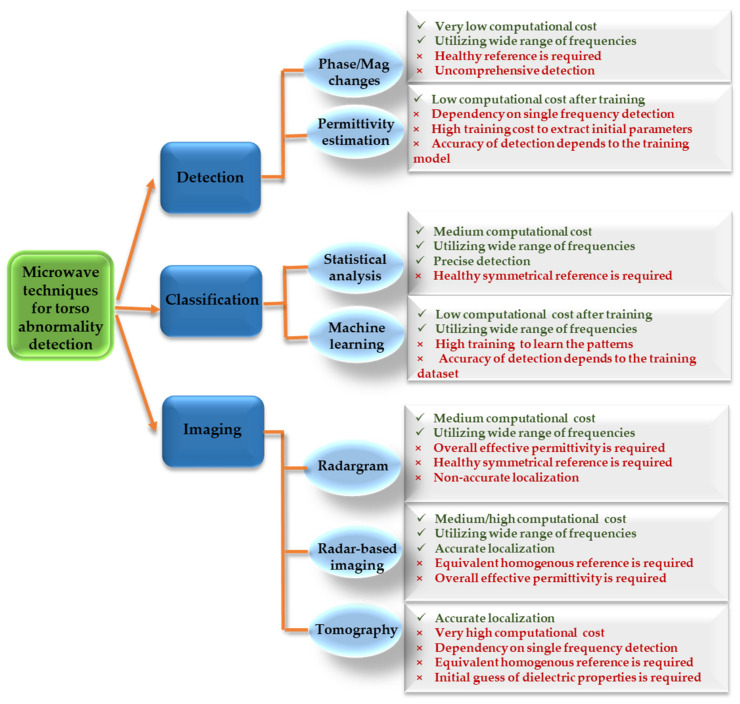
An overview of EM thoracic abnormality detection techniques.

These changes can be used to detect/classify the abnormality and/or localize it by creating an image from the investigated domain. This image can be created either by calculating the scatter fields inside the domain (radar-based imaging) or by calculating dielectric properties of the tissues (tomography). Based on processing outcome, EM processing techniques can be classified into three main categories: (1) detection only methods, (2) detection and classification methods, and (3) detection and localization (imaging) methods. Figure 16 presents an overview of the current microwave techniques. In this section, each technique applied to the detection of different thoracic diseases is explained in detail. Then, the advantages and disadvantages of each method are discussed and compared in terms of practicality, computational time and accuracy.

### 5.1. Detection Only Methods

These methods aim at detecting any abnormality within the torso without providing any information on the location of that abnormality. Hence, their computational time is lower than localization techniques. These techniques are more suitable in disease detection where the location of abnormality is not important such as, hepatic steatosis or bronchial asthma detection. In this section, different methods utilized in malignancy detection are reviewed. These methods can be classified into two main categories based on their detection approach. First group exploits the change in magnitude and/or phase of electromagnetic signals. Second group estimates the overall effective permittivity of the medium.

#### 5.1.1. Phase/Magnitude Changes

Some techniques compare the magnitude or phase of the propagation coefficient between control and diseased group to achieve a distinguishable trend [9,70,71,81,82,83,84,85]. These techniques are mostly utilized in linear scanning platforms operating in a mono-static or bi-static (multi-static with use of two antennas) data acquisition mode. The assessment of low and high frequency regions of reflection or transmission coefficients results in a contrast between measured healthy and unhealthy signals.

**Figure 17:**
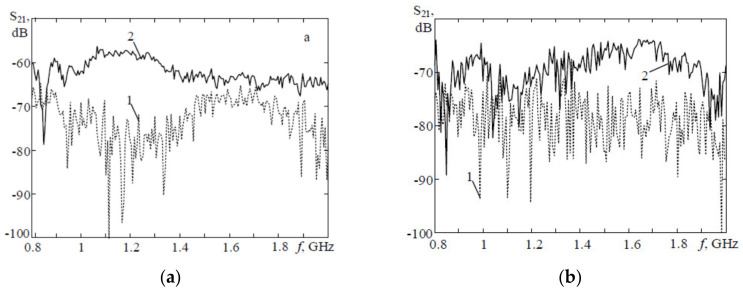
Propagation coefficient in a brachial asthma subject (solid line) compared to a healthy case (dashed line): (**a**) scan of upper part of lungs; (**b**) scan of lower part of lungs. Reproduced with permission from [82]. Copyright 2017. IEEE.

In [82], propagation coefficients of upper and lower parts of the lungs are compared between 12 patients with confirmed diagnosis of brachial asthma and a healthy controlled group comprised of 10 individuals with appropriate age and sex, matched with patients by morphological characteristics of the chest. Analyses of measured signals from patient group shows that the signals experience significantly lower attenuation compared to the measured signals from healthy controlled group in the bandwidth from 0.9 to 1.5 GHz when propagating through the upper part of the lungs (see Figure 17a). However, scanning of lower parts of the lungs show maximum different between healthy and controlled group at higher frequency bandwidth from 1.2 to 2 GHz, (see Figure 17b). Consequently, monitoring of propagation coefficient at these frequency regions provides higher accuracy of disease detection.

In [81], the phase of transmitted signals at a single frequency is used to create a phase diagram of the chest to detect any inhomogeneity within lungs. In [83], the transmission coefficient is converted into a corresponding voltage at the output and the diagram of the output voltage is created to detect any inhomogeneity inside the chest area. It is also shown that the maximum probability of detection with more precise borders takes place at higher frequencies.

**Figure 18:**
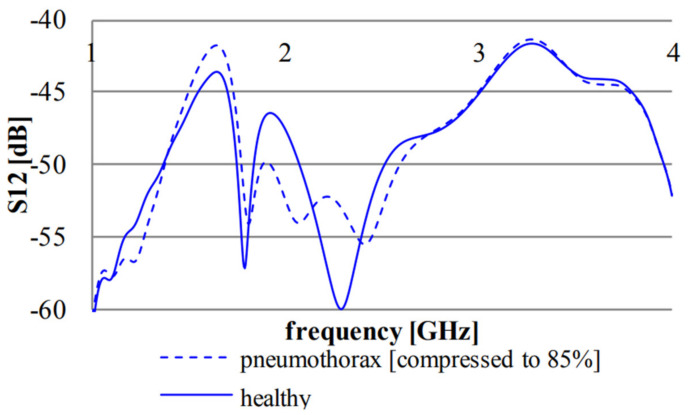
Magnitude of transmission coefficient in healthy and pneumothorax scenarios. Reproduced with permission from [70]. Copyright 2014 IEEE.

**Figure 19:**
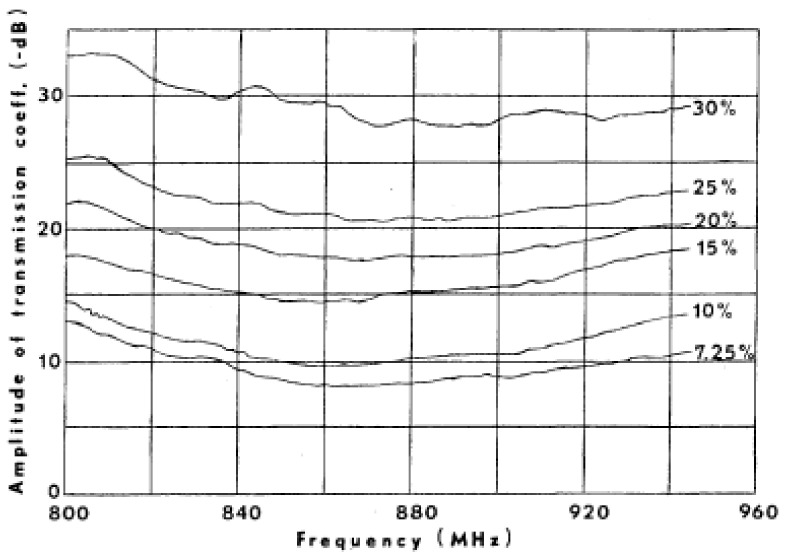
Increase in magnitude of transmission coefficients with increased percentage of accumulated fluid in lungs. Reproduced with permission from [9]. Copyright 1978 IEEE.

The magnitude of transmission coefficients is also exploited to detect pneumothorax [70] or pulmonary edema [9]. It is shown that the maximum difference between transmission coefficients in healthy and pneumothorax scenarios occurs at frequency of 2.3 GHz and is about 7.1 dB, (see Figure 18). The existence of the trapped air in the chest cavity due to the disease results in changes in electric field distribution at this frequency. Additionally, it is shown that the magnitude of transmission coefficients increases in the bandwidth 800–955 MHz as the result of increase in lung water volume [9] (see Figure 19).

Furthermore, changes in the magnitude of reflected signals is exploited to detect the presence of tumors in lungs [84]. Differences between the measured signals with and without presence of tumor are used for detection purposes. The differences between healthy and unhealthy signals were mostly found at frequencies below 6 GHz and changing the size of the tumor in the lung, creates a shift in the magnitude of reflection coefficients [85].

Although phase or magnitude of electromagnetic signals might differ in unhealthy torso due to the existence of any abnormalities or disease, this difference cannot lead to a comprehensive and accurate detection of the disease. For example, as realized from the results in [82], the frequency at which maximum contrast occurs might vary depending on the used sensors (antennas) and size of the torso. Hence, this contrast is not always an indicator of the disease or abnormality.

#### 5.1.2. Effective Permittivity Estimation

Some approaches try to estimate the overall effective permittivity of the torso to detect changes in permittivity due to presence of an abnormality. These methods usually model the overall effective permittivity of torso as a function of transmission coefficients. Then, the weight parameters for the model are extracted using training process [13,86]. This model can be linear [13] or non-linear based on spatial statistical technique [86]. In the spatial technique, the effective permittivity of torso from the receiver perspective is modeled using a variogram. This is related to spatial dependence of each signal, a vector of quadric regression function, and a vector of regression coefficients. Variogram is calculated for each receiver using the measured S-parameters at that receiver due to transmission from antennas located at a determined neighboring antenna. To estimate the effective permittivity at each receiver, the best unbiased estimation regression coefficients is obtained by training the model using training samples. These training samples are generated by measured S-parameters of the homogenous equivalent medium with different known permittivity values. After finding the regression coefficients vector by training, it can be used to estimate the effective permittivity of any test medium.

**Figure 20:**
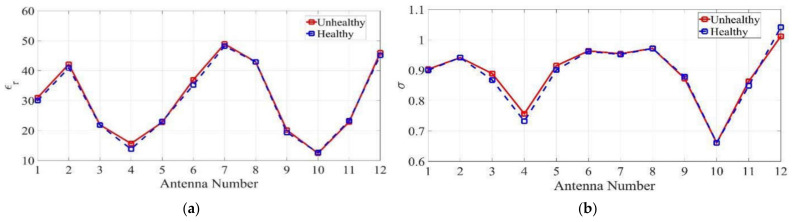
(**a**) Estimated permittivity and (**b**) conductivity from viewpoint of various antennas around torso for healthy and unhealthy lung cases. Reproduced with permission from [86]. Copyright 2017 IEEE.

**Figure 21:**
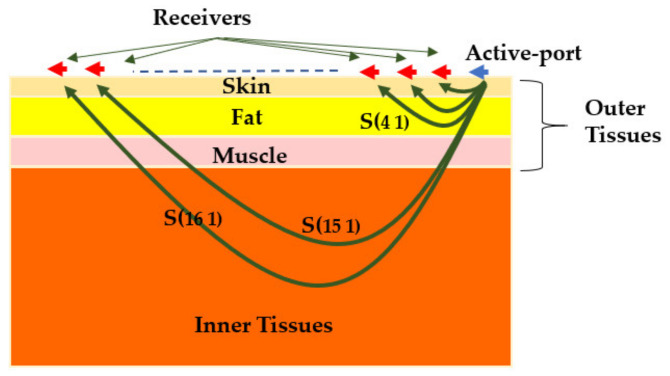
Permittivity estimation of inner layer (lung) by modeling it as a weighted summation of S-parameters (concept taken from [13]).

In [86], the spatial statistical technique is used to estimate the effective permittivity and conductivity from the viewpoint of each antenna. To do so, multi-static signals are collected in a circular platform for healthy lung and lung with a tumor. The estimated permittivity and conductivity values in unhealthy scenario are slightly higher than the healthy one due to existence of tumor (see Figure 20). Although, it cannot directly interpret the health status of the lung by these estimated values, they can be used as prior information in radar-based imaging to improve the quality of the image in localizing the tumor. In [13], a wearable health monitoring sensor for detection of pulmonary edema is proposed. The 16-port sensor is placed on the human chest to detect any lung abnormalities by estimating the effective permittivity of lung (see Figure 21). Therefore, the effective permittivity of lung at a single frequency is expressed as a weighted summation of S-parameters measured at each port. The parameters of the weight vector are set based on training the model by assigning different dielectric characteristic to the inner tissue (lungs). Using multiple ports mitigate the effect of the outer layer (skin, fat, and muscle) on lung’s permittivity and allow to characterize the inner layer tissue. The validation of this technique on measurements of the healthy and edema lung shows less than 11% error in the calculated permittivity of lung compared to the measured value. It is observed that the unhealthy lung has a higher permittivity value than the healthy lung due to accumulated water inside the unhealthy lung.

Permittivity estimation methods reveal promising results in detecting lung abnormalities. However, the performance is hindered by training requirement to determine coefficient vectors and achieve accurate result. Hence, they may not achieve reliable results when the test models are considerably different from the training model, which is the case in clinical application.

### 5.2. Detection and Classification Methods

These methods aim at classifying and labelling the collected data as healthy or unhealthy based on their underlying pattern and characteristics. They can be classified into two main categories: the first group exploits symmetry of the torso in a statistical approach, whereas the second group utilizes artificial intelligence techniques to differentiate between healthy and unhealthy torso.

#### 5.2.1. Statistical Analysis

Multivariate energy statistics method is another detection technique that operates based on the assumption that organs within the left and right sides of torso have close dielectric properties [45]. Hence, the changes in the dielectric properties of unhealthy tissue enhance the contrast with surrounding tissues. Therefore, the similarity of electromagnetic signals collected from left and right sides of torso is reduced.

In [24], a wearable electromagnetic belt is used to detect hepatic steatosis using multivariate energy statistics method. Any changes in the dielectric property of liver due to excess fat in hepatic steatosis increase the contrast between the liver and surrounding tissues. The method calculates the distance correlation of the measured transmission coefficients between symmetric paths from the left and right sides of torso. The results indicate a peak measured dissimilarity of 15.1% between transmission coefficients of left and right sides of the torso in steatotic liver, which is much higher than the healthy case [24]. Hence, healthy and steatotic liver can be classified based on the left/right permittivity contrast after determining a threshold for healthy liver.

The limitation of this technique is the requirement of almost symmetrical setup to achieve a reliable classification. Additionally, a sufficient number of healthy signals is required in order to define the threshold and set the boundary between healthy and unhealthy signals.

#### 5.2.2. Machine Learning

Supervised machine learning is a form of artificial intelligence technique, which can be used in classifying healthy and unhealthy cases. This method requires a set of training signals to learn the characteristics of input signals. Then, the trained model is validated on test signals. Supervised machine learning framework is utilized to learn an inferring model for hepatis steatosis from the data collected by an antenna operating across 0.4–1 GHz bandwidth in a mono-static mode [27]. Data are collected from a simulated numerical torso by changing the permittivity of the liver from 30–60 within the frequency range of the used antenna. Real and imaginary parts, magnitude and phase of the collected S-parameters are used as inputs for the supervised classifier. Labels of healthy and unhealthy liver are assigned based on the permittivity of liver. Learning is performed using different classification techniques and leave-one-out-cross-validation is used to evaluate the classification performance. The results indicate that this system can detect hepatic steatosis with accuracy of more than 97% for the simulated torso model.

The main drawback of this method is the requirement for enough number of training signals from various stages of disease. The accuracy of this method is highly dependent on the training data base. Insufficient and non-comprehensive training set results in misclassification.

### 5.3. Detection and Localization Methods

These methods aim at finding the location of abnormality within the torso by forming an image of the investigated domain. This is achieved either by calculating the scattered fields or by calculating dielectric map of the investigated domain. The target, which can be tumor, fluid, or fat infiltration, can be recognized by its high intensity or dielectric contrast with surrounding tissues. Based on the utilized approach in creating the image, the localization methods can be categorized into three main groups: (1) radargram, (2) radar-based imaging, and (3) tomography. In this section, an overview of each approach is presented. All these imaging techniques require an equivalent homogenous medium for detection and localization. The difference of measured signals with and without of the investigated domain is considered in the calculations.

#### 5.3.1. Radargram

**Figure 22:**
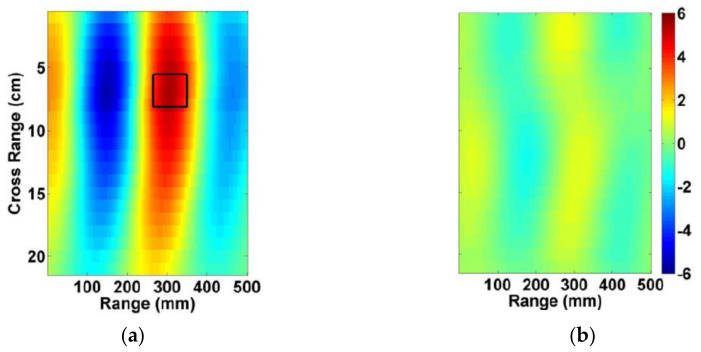
Radargram of (**a**) unhealthy and (**b**) healthy lungs. The square shows the location of 10 mL water inside the lung. Reproduced with permission from [26] (open access).

This technique provides a two-dimension visualization of torso by analyzing the reflection coefficients [26,56,58,72]. This method transfers measured signals to time domain using an inverse Fourier transform. Then, using the wave speed in the medium the time domain signal is scaled to show the intensity of the reflected signal within an investigated depth. Finally, each of the measurement are overlaid to create the radargram (image). Usually, the average permittivity of torso tissues is used to define the propagation speed. Hence, the target is highlighted in the image due to its variant permittivity. In [26], radargram technique is used for early detection of congestive heart failure due to fluid accumulation inside lungs. The rear side of torso is scanned using one antenna in a linear platform and a mono-static data acquisition mode. The symmetry of left and right lungs is exploited to detect and localize the target (see Figure 22).

**Figure 23:**
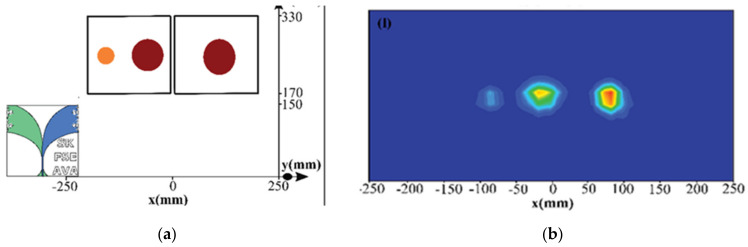
(**a**) Simulated lungs with tumors; (**b**) created image by TDR data analyzing. Reproduced with permission from [56]. Copyright 2020 IEEE.

In [56], time domain reflectometer (TDR) data, obtained in a linear platform, are used to create an image of the scanned area. The imaging algorithm consists of pre-processing and quadratic envelop detection. The pre-processing step reduces background noise and clutters using absolute function and Shannon energy, which calculates the average energy spectrum of the signal. Then, local maximums of the signal are derived using an envelope function to obtain shape of the tumor. Finally, the fourth order quadradic function is applied to enhance the edge detection of tumor. Figure 23 shows the created image of lungs with three tumors in right and left lungs.

The main limitation of this technique is its insensitivity to deeper malignant tissues that are far away from the antenna. Hence, its accuracy in localization small and deep targets is low. Additionally, it needs the average effective permittivity of tissue to define the propagation speed. This limits the suitability of the technique in clinical applications.

#### 5.3.2. Radar-Based Imaging

Radar-based imaging techniques can be classified as time-domain confocal microwave imaging [20,21,50] and fast frequency-based radar imaging [18,19,29,38,77,87]. Both approaches require a calibration step when the free-space antennas are utilized to scan the torso. This step is necessary to reduce the significant reflections from air-skin interface which mask the desired target reflections. To calibrate the measured signals, the average of all measured signals is subtracted from each measured signal. As antennas are located at the same distance from skin, the effect of air-skin reflections is almost similar in all measurements. Calibrated signals are then used in further calculations to localize and detect the malignant tissue.

Time-domain confocal microwave imaging has broadly been used in head and breast imaging and demonstrates successful results in tumor or torso fluid detection [20,21,50]. The method uses an ultra-wideband signal to illuminate the imaging object, whereas the received signals in time domain are used to create a map of scattered fields inside the investigated domain. Assuming the focal points inside the imaging domain as point scatterers, the received signals are delayed depending on the wave traveled distances from each transmitter to the individual point scatterers and the receiver. The sum of the delayed signals at all the focal points are then used to calculate the scattered energy and obtain the final image.

**Figure 24:**
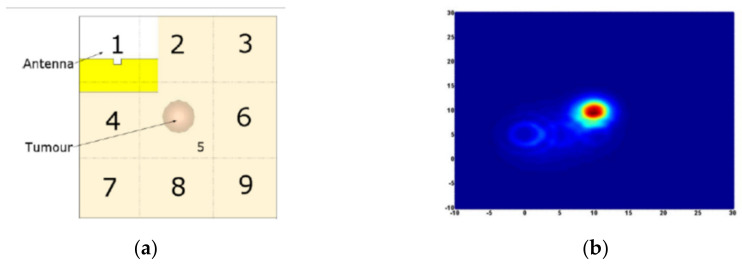
(**a**) Front view of the locations of the antenna and tumor; (**b**) created image with confocal imaging after smoothing. Reproduced with permission from [20]. Copyright 2014 IEEE.

In [20], the human torso is modeled as a 100 × 100 × 70 mm block in which different layers (skin, fat, muscle, bone, and lung) are presented with their dispersive dielectric properties. The difference between reflection coefficients with and without the presence of tumor is used for confocal imaging. A smoothing process is performed on signals to improve the quality of the resultant image by applying a filter which provides nonparametric smoothing of signals peaks and filter the noise. Using this filter, the detection and localization of the tumor is enhanced (see Figure 24). In [21], confocal imaging of human chest model using the reflection coefficients reveals better tumor detection and localization during exhale.

Fast frequency-based radar imaging is used in several microwave systems to detect and localize tumor [19] or fluid [18,29,38,77,87] inside torso. This method first calibrates measured signals, which are the difference in S-parameters between presence and absence of torso, using average subtraction techniques to remove artifacts at each frequency step. Then, the calibrated data are multiplied by a back-propagation Green’s function and summed over all antennas’ positions to calculate intensity of electromagnetic fields at each scatter position inside torso. The Green’s function is modeled as the multiplication of first order first kind Bessel function and an exponential term. Both Bessel and exponential functions are dependent to the wave path distance and wave number.

**Figure 25:**
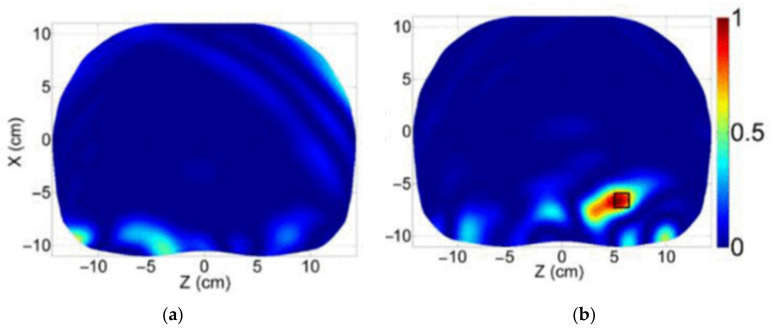
Reconstructed image of a (**a**) healthy lung, and (**b**) edema lung with accumulated fluid using fast-frequency radar-based imaging method. Reproduced with permission from [18] (open access).

In [18,19], fast-frequency radar based imaging is performed to detect and localize abnormality inside lungs. Figure 25 present the detection and localization of accumulated fluid in lung using this technique.

**Figure 26:**
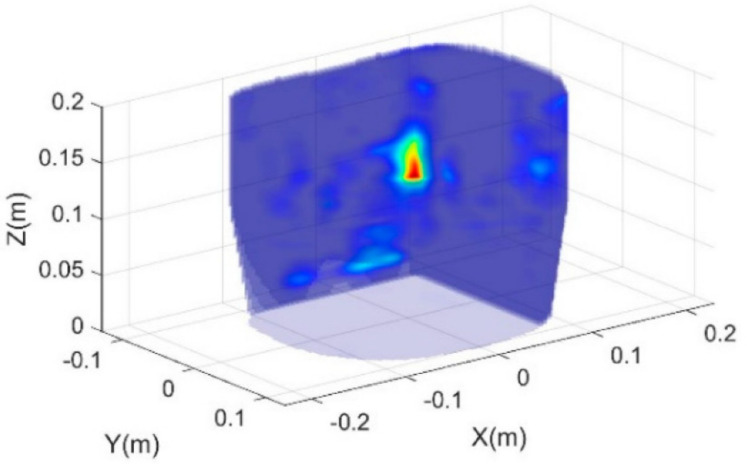
Three-dimensional (3D) image of the torso with accumulated water in the lung. Reproduced with permission from [33] (open access).

In [33], fast-frequency imaging in conjunction with slice interpolation technique is used to create a 3D visualization of torso. To do this, different slices along torso are scanned with 1 mm resolution using a mechanically moveable array of antennas located on a flange. This system could accurately determine the location and volume of the embedded water in a phantom lung (See Figure 26).

Radar-based imaging techniques need a prior information about the effective permittivity value of the medium. This value is usually set based on the average permittivity of tissues at mid-point frequency. However, in clinical application the average effective permittivity may be variant in different individuals who have different body shapes and habitus. Hence, effective permittivity estimation technique is usually needed to estimate the overall effective permittivity of the medium prior to imaging.

#### 5.3.3. Tomography

Microwave tomography is based on the fact that biological tissues have different dielectric properties at microwave frequencies and thus they can be imaged by solving an inverse and forward scattering problem. In this method, the measured electric fields are used as the input of an inverse problem derived from Maxwell’s equations. The non-linear invers problem is ill-posed as there is more than one solution to satisfy the equations. Hence, additional information and assumption are required to reach the correct solution. Optimization-based methods [88,89,90], born iterative methods [91,92], and Newton-based methods [93,94,95] are mostly exploited to solve the inverse problem in an efficient way in at iteration mode. At each iteration, the measured field and calculated field with current dielectric distribution are compared and the error is estimated. The solution is found when the calculated error is reached to a predefined threshold. This technique requires a priori information of dielectric properties of investigated domain to achieve reliable results. Torso microwave tomography is not as a common as breast and head tomography. This is due to the large size of the human torso, which increases the number of parameters in calculations, resulting in high computational costs and less probability of a successful convergence of the solution.

**Figure 27:**
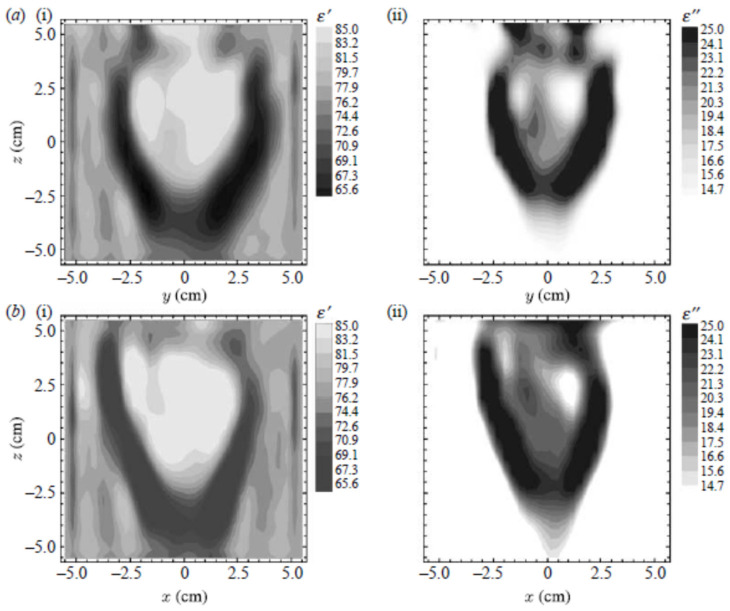
Reconstructed image of canine heart at 2.4 GHz at two cross-sections using microwave tomography (**a**) X = 1.5 cm, (**b**) Y = 1.5 cm. Reproduced with permission from [23]. Copyright 2000 IEEE.

Microwave tomography is utilized to detect and localize lung cancer [96,97] or create an image of intact canine or swan heart [23,98]. In [97], the method is applied to two-dimensional computer-simulated model of the chest with three high contrast inhomogeneities which model lung tumors. The location of the tumors is successfully detected using the tomography approach. In [23], a three-dimensional gradient method is used to reconstruct a dielectric map of an excited static canine heart at frequency of 2.4 GHz. The resultant image, presented as 2-D vertical cross sections, reflects the structure complexity of the heart well, (See Figure 27). The reconstructed dielectric properties are close to the dielectric properties of myocardium and left/right ventricles are obvious in the reconstructed image.

Three-dimensional microwave tomography has also shown successful results in the reconstruction image of infracted canine heart [99]. The reconstructed images show both the shape of the heart and the region of infraction which is highly correlated with anatomical slices. Hence, microwave tomography might have potential for both structural and functional cardiac imaging.

**Figure 28:**
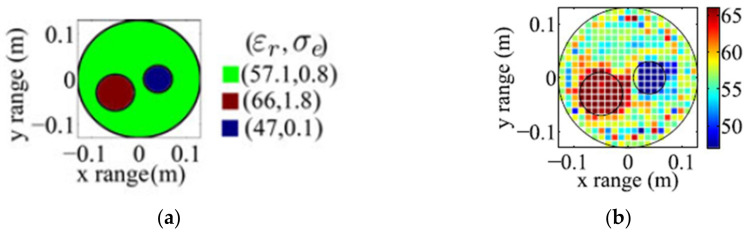
(**a**) Simulated circular inhomogeneous phantom; (**b**) reconstructed image of the relative permittivity using microwave tomography. Reproduced with permission from [100]. Copyright 2014 IEEE.

Capability of the in-body microwave tomography has also been tested using a circular heterogenous phantom generated by FDTD simulations [100]. The radius of the phantom is 13 cm with two more embedded circular cylinders to create inhomogeneity. The 2-D microwave tomography at 403.5 MHz using Newton-based method showed successful result in detection and localization of the inhomogeneities within the simulated torso, (See Figure 28).

**Figure 29:**
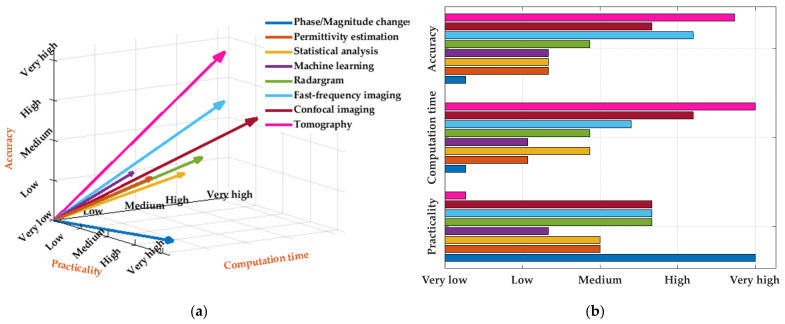
Comparison between EM thoracic scanning techniques based on their complexity, computation time, and accuracy; (**a**) 3-D representation; (**b**) bar graph representation.

Practicality, computation time, and accuracy of all microwave detection techniques is compared in Figure 29. Practicality is defined based on the required system configuration and its pre-requisites. As seen from this figure, there is always a compromise between practicality, computation time and accuracy of the utilized methods. For instance, tomography methods, provide the most accurate detection decision, but they have the most impractical hardware setup and require the highest computational time. On the contrary, the detection only methods, such as magnitude/phase monitoring, has the simplest setup, but also achieves the lowest accuracy. Therefore, depending on the required accuracy and system cost, a suitable configuration might be selected.

## 6. Conclusions

A comprehensive review of EM torso scanning systems has been presented. Different data acquisition methods, scanning platforms, antenna types and detection/imaging algorithms are reviewed and categorized. It is shown that the idea of detecting malignancies inside torso using microwave signals has advanced significantly since its introduction in 1973. The systems have advanced from detecting the malignancy only to mapping its exact location and dimension. New detection techniques are developed thanks to significant advancements in computational capacities and incorporation of different statistical techniques and machine learning methods. This promises more accurate and real-time detection decisions that can be used as a complementary technique besides conventional imaging devices. Additionally, it strengthens the confidence in EM torso scanning systems as powerful tools for monitoring purposes and onsite diagnosis, which are the major limitations of existing imaging systems. As can be realized from Table 1, the higher accuracy comes with increased system’s complexity and computational cost. This creates an exciting challenge and the future research roadmap, where solutions are needed to maintain accuracy of EM systems while simplifying their design and computational cost. While the future is unknown, it is guaranteed that only systems that can satisfy these two factors will have the competing edge in clinical settings.

## Figures and Tables

**Table 1:** Summary of available torso scanning systems.

Ref.	System Configuration	Antenna	Algorithm	Advantages	Disadvantages
[34]	Linear array of antenna in multi-static data acquisition mode	Unidirectional wideband free space 3-D loop-monopole antenna	Fast frequency imaging	•High accuracy of detection and localization •High practicality •Medium computation time	•Requirement of healthy symmetrical reference •Requirement of average permittivity of tissues •Medium penetration
[26,35]	Linear array of antenna in mono-static data acquisition mode	Unidirectional wideband free space folded antenna	Radargram	•High practicality •Medium computation time	•Requirement of healthy symmetrical reference •Requirement of average permittivity of tissues •Not suitable for deep target detection •Medium penetration
[27]	Mono-static data acquisition mode	Unidirectional wideband on-body matched waveguide antenna	Machine learning	•Low computation time after training •Simple structure	•Requirement of training •Low practicality •Not suitable for deep target detection
[29]	Circular array of antenna in multi-static data acquisition mode	Unidirectional wideband free space metamaterial unit-cell loaded Yagi-antenna	Fast frequency imaging	•High accuracy of detection and localization •High practicality Medium computation time	•Requirement of average permittivity of tissues •Medium penetratio
[31,32,70]	Bi-static data acquisition mode	Omni directional narrowband on-body matched antenna	Phase/Mag changes	•High practicality •Low computation time	•Low accuracy •Low penetration
[33]	Circular array of antenna in multi-static data acquisition mode	Unidirectional wideband free space resonance-based reflector antenna	Fast frequency imaging	•High accuracy of •High practicality •Medium computation time	•Requirement of average permittivity of tissues •Medium penetration •Complex setup
[36]	Circular array of antenna in mono-static data acquisition mode	Unidirectional wideband free space loop- dipole antenna	Fast frequency imaging	•High accuracy •High practicality •Medium computation time	•Requirement of average permittivity of tissues •Medium penetration •Not suitable for deep target detection
[38,59,87]	Quasi-circular antenna in mono-static data acquisition mode	Unidirectional wideband free space pattern reconfigurable metasurface antenna	Fast frequency imaging	•High accuracy •High practicality •Medium computation time •High penetration	•Requirement of average permittivity of tissues
[24]	Circular antenna in multi-static data acquisition mode	Unidirectional wideband on-body matched loop-dipole antenna	Statistical analyses	•Medium practicality •Medium computation time	•Requirement of a symmetric healthy part •Low-medium accuracy •Requirement of healthy threshold •Medium penetration
[86]	Circular antenna in multi-static data acquisition mode	Unidirectional wideband free space loop-dipole antenna	Permittivity estimation	•Medium practicality •Low computation time •Suitable for enhancing radar-based imaging	•Requirement of training •Low-medium accuracy •Medium penetration
[21,56]	Linear scanning monostatic data acquisition mode	Unidirectional wideband free space Vivaldi antenna	Radargram	•High practicality •Low computation time	•Not suitable for deep target detection •Medium penetration
[21]	Linear scanning mono-static data acquisition mode	UWB Antenna Free Space Antenna	Confocal imaging	•High practicality •Low computation time	•Low accuracy •Requirement of average permittivity of tissues •Not suitable for deep target detection •Low penetration
[23,98,101]	Quasi-circular antenna in multi-static data acquisition mode	Dielectric Loaded Waveguide	Tomography	•High accuracy •High penetration	•Low practicality •Very high computation time

## Data Availability

Not Applicable.
